# Mindfulness-Based Interventions for Chronic Pulmonary Diseases: A Systematic Review of Effects on Anxiety, Depression, Stress, Dyspnea, and Quality of Life

**DOI:** 10.3390/bioengineering12090931

**Published:** 2025-08-29

**Authors:** Alessia Bramanti, Colomba Pessolano, Marina Garofano, Angelantonio Maglio, Michele Ciccarelli, Luana Budaci, Mariaconsiglia Calabrese, Andrea Marino, Francesco Loria, Francesco Corallo, Placido Bramanti, Carmine Vecchione, Alessandro Vatrella

**Affiliations:** 1 Department of Medicine, Surgery and Dentistry, University of Salerno, Via S. Allende, 84081 Baronissi, Italy; abramanti@unisa.it (A.B.); amaglio@unisa.it (A.M.); mciccarelli@unisa.it (M.C.); macalabrese@unisa.it (M.C.); andremarino@unisa.it (A.M.); cvecchione@unisa.it (C.V.); avatrella@unisa.it (A.V.); 2 Azienda Ospedaliero-Universitaria “San Giovanni di Dio e Ruggi d’Aragona”, Via San Leonardo, 84125 Salerno, Italy; lu.budaci@gmail.com (L.B.); francescoloria94@gmail.com (F.L.); 3 IRCCS Centro Neurolesi Bonino-Pulejo, Via Palermo, S.S. 113, C.da Casazza, 98124 Messina, Italy; francesco.corallo@irccsme.it; 4 Faculty of Psychology, Università degli Studi eCampus, Via Isimbardi 10, 22060 Novedrate, Italy; bramanti.dino@gmail.com

**Keywords:** mindfulness-based interventions, chronic pulmonary disease, virtual reality, psychological distress, quality of life

## Abstract

(1) Background: Chronic pulmonary diseases (CPDs), such as COPD, asthma, and interstitial lung disease, are often accompanied by psychological distress and reduced quality of life. Mindfulness-Based Interventions (MBIs), including digital and virtual reality (VR) formats, have emerged as promising non-pharmacological approaches to improve symptom management and well-being. This systematic review aimed to evaluate the effectiveness of MBIs—delivered in-person or digitally—on anxiety, depression, disease-related stress, dyspnea, and health-related quality of life in individuals with CPDs. (2) Methods: A systematic review was conducted following PRISMA guidelines across PubMed, Scopus, and Web of Science (2005–2025). Thirteen studies (8 randomized controlled trials, 5 non-randomized) met inclusion criteria. Outcomes assessed included psychological distress, physical symptoms, and health-related quality of life (HRQoL). Risk of bias was evaluated using RoB 2 and ROBINS-I tools. (3) Results: MBIs were associated with improvements in anxiety, depression, stress, and HRQoL in several studies. Interventions were generally well-tolerated and effective across various delivery methods, including digital and VR-based formats. Improvements were especially notable when interventions were tailored and sustained. (4) Conclusions: MBIs, including digital and VR-delivered formats, show promise in supporting psychological and physical outcomes in CPD populations. They represent a feasible and complementary tool in modern respiratory care.

## 1. Introduction

Chronic pulmonary diseases (CPDs)—including chronic obstructive pulmonary disease (COPD), interstitial lung disease (ILD), and chronic asthma—are progressive, debilitating conditions that affect millions of people worldwide and are associated with a significant physical and psychological burden [1]. In addition to core respiratory symptoms such as dyspnea, fatigue, and reduced exercise tolerance, many people with CPDs suffer from high levels of anxiety, depression, and health-related distress [2,3]. Psychological symptoms are not only secondary to physical illness but can exacerbate symptom perception, reduce self-management, and negatively affect clinical outcomes [4]. Therefore, there is growing recognition of the need for integrative, biopsychosocial approaches to the treatment of chronic respiratory diseases.

Among non-pharmacological strategies, mindfulness-based interventions (MBIs) have proven to be promising adjuncts in the management of chronic diseases. MBIs are rooted in contemplative traditions and have been operationalized in Western clinical psychology through interventions such as Mindfulness-Based Stress Reduction (MBSR) and Mindfulness-Based Cognitive Therapy (MBCT). MBIs are structured programs that teach individuals to develop non-judgmental, present-moment awareness [5]. Theoretical models suggest that mindfulness promotes psychological well-being through mechanisms such as improved attentional control, emotional regulation, interoceptive awareness, and decentering, allowing individuals to observe thoughts, emotions, and sensations without reactivity or avoidance [6,7].

In patients with CPDs, MBIs may be particularly relevant as they can help sufferers develop a more accepting relationship with unpleasant sensations such as breathlessness, reduce catastrophic thinking, and improve mood and quality of life (QoL) [8,9]. Several studies have shown that traditional, face-to-face MBIs can lead to a reduction in anxiety and depression, an improvement in the perception of breathlessness, and improved coping. However, these programs typically require weekly attendance, physical travel, and sustained effort over several weeks—barriers that may limit access or participation for individuals with limited mobility, fatigue, or rural residence.

In response to these limitations, technology-enabled methods, particularly virtual reality (VR), have been explored as a way to broaden access and increase engagement. VR-based MBIs use immersive, computer-generated environments—often replicating calming natural environments—to guide mindfulness practice with rich sensory input and spatial presence [10]. Preliminary research suggests that VR can improve attentional focus, reduce distraction, and increase emotional engagement, potentially enhancing the effects of mindfulness practice [11,12].

Beyond accessibility, VR may be uniquely suited for CPD patients because of its potential for physiological modulation (e.g., immersive calming environments that can influence autonomic activity and alter the perception of breathlessness), enhanced attentional focus (reducing external distractions and supporting mindfulness practice in the presence of fatigue or dyspnea), multisensory engagement (reinforcing interoceptive awareness and breathing regulation), and improved adherence (increasing motivation and reducing dropout, particularly among those with mobility limitations) [13,14,15,16,17].

Nevertheless, the current evidence on MBIs in CPDs is heterogeneous and fragmented: most studies focus on COPD, with limited data available for asthma and ILD; comparability is poor due to variability in study design, outcomes, and patient populations; and no systematic synthesis has examined different delivery formats (in-person vs. digital) in a single review. While VR is discussed in the broader literature as an innovative delivery mode, none of the studies included in this review actually used VR, leaving its clinical impact in CPDs unexplored. We acknowledge that COPD, ILD, and asthma differ in symptom profile and disease course, but they share persistent symptoms such as dyspnea and fatigue, high prevalence of psychological distress, and the need for long-term self-management. MBIs target transdiagnostic mechanisms—such as attentional control, emotion regulation, and acceptance of bodily sensations—that are relevant across these conditions, making pooled analysis valuable for identifying cross-cutting benefits while noting phenotype-specific differences.

To the best of our knowledge, previous systematic reviews on psychological interventions in COPD or MBIs in other chronic illnesses have not included asthma and ILD in the same synthesis, have not compared delivery formats, have not discussed the potential role of VR in CPDs, and in some cases have referred only generically to “relaxation techniques” without specifying structured mindfulness-based approaches [9,18,19,20,21,22]. Our review is the first to integrate these elements into a single, comprehensive analysis.

This systematic review therefore aims to summarize the current evidence on MBIs for people with chronic lung disease, examining their effects on psychological outcomes (e.g., anxiety, depression, stress), physical symptoms (e.g., dyspnea, fatigue), and health-related quality of life (HRQoL). In addition, feasibility, acceptability, and engagement in different forms of delivery are explored, and the potential role of VR is discussed as a promising future direction for research and practice in respiratory medicine.

## 2. Materials and Methods

### 2.1. Study Protocol

The study protocol was designed in accordance with the PRISMA guidelines (Preferred Reporting Items for Systematic Reviews) [23], a rigorous methodological approach prior to the literature search and data synthesis with a specific focus on the research questions and the clinical effectiveness of mindfulness-based interventions (MBIs), delivered with or without VR. The analysis focuses on the effects of these interventions and their role in improving psychological distress, reducing anxiety, depression, low mood, disease-related stress, and other health-related outcomes, evaluating their impact on self-management of symptoms and HRQoL. Before starting the june and data synthesis, the related study protocol was registered in the International Prospective Register of Systematic Reviews (PROSPERO) database of systematic reviews (https://www.crd.york.ac.uk/PROSPERO/view/CRD420251072411, accessed on 29 May 2025). The formulation of the research question and the strategy and criteria for study selection were developed using the PICO model [24]. The study question focused on the following:

- P (Population): Patients with chronic respiratory diseases (e.g., COPD, pulmonary fibrosis, asthma, etc.)
- I (Intervention): Psychological intervention combined with mindfulness, where mindfulness is delivered with or without VR
- C (Comparison): Psychological intervention alone or no intervention
- O (Outcomes): QoL, anxiety, disease-related stress, any healthcare outcome

### 2.2. Search Strategy and Study Selection

The literature search was conducted electronically across three databases: PubMed, Scopus, and WOS. The database screening was carried out independently by three reviewers (MG, CP, AB) covering studies published from January 2005 to 1 June 2025, which represents the time frame considered for this review, using the following keywords combined with Boolean operators: Chronic respiratory disease, COPD, chronic obstructive pulmonary disease, mindfulness OR mindfulness meditation, virtual reality, VR, avatar, psychological intervention, psychosocial intervention. Complete search strategies are provided in Appendix A. The collected citations were recorded, duplicates were eliminated using EndNote, and the titles and abstracts obtained were independently screened by two reviewers (MG, CP). The full texts of potentially relevant papers and ambiguous abstracts were reviewed independently by the same authors (MG, CP), who resolved disagreements through discussion and consensus and, if necessary, with the involvement of a third reviewer (AB). The inclusion criteria were as follows:

- Source: studies published in the English language from 2005 to 1 June 2025;
- Study design: randomized controlled trial (RCT), observational studies, feasibility studies, and qualitative studies;
- Study population: Adults (>18) with CPDs
- Study intervention: MBIs delivered with or without VR;
- Study outcomes: MBIs are useful for clinicians to reduce stress, disease-related psychological distress and suffering, improving QoL or other healthcare outcomes in the sample of patients with chronic pulmonary diseases.

The exclusion criteria were as follows:

- Source: studies published before 2005 or after 1 June 2025;
- Study intervention: Studies not involving MBIs, other systematic reviews, studies utilizing other psychological interventions, or interventions focusing on pharmacological or surgical treatments.
- Study outcomes: studies not reporting psychological outcomes; studies without clinical outcomes related to stress reduction, healthcare impact, or QoL.

### 2.3. Data Extraction and Collection

Eligible studies were included or excluded upon mutual agreement. In cases of disagreement regarding a manuscript’s inclusion based on abstract evaluation, discrepancies were resolved through discussion and consensus. If consensus was not reached, a third reviewer (AB) was consulted for the final decision. The data extraction process followed established methodologies and was structured to align with the research questions of this review. Extracted data included: (a) author, year and country; (b) study design; (c) sample size (population, mean age, % male); (d) intervention group; (e) control group; (f) main outcomes; (g) secondary outcomes; (h) key findings (Table 1). This systematic approach ensured a rigorous and consistent collection of relevant data, facilitating a comprehensive synthesis of evidence to address the research questions. 

#### Quality Assessment

The risk of bias for the studies included in this systematic review was independently evaluated by two reviewers (MG, AB). In cases of disagreement, a third reviewer (CP) was consulted to facilitate discussion and reach a consensus. For RCTs, the Cochrane Risk of Bias tool (RoB 2) [37] was applied, assessing five key domains: randomization process, deviations from intended interventions, missing outcome data, outcome measurement, and selection of the reported results. Each domain was rated as having a “low”, “high”, or “unclear” risk of bias (Table 2).

For non-randomized studies, the ROBINS-I tool [38] was employed to assess potential bias across several domains: confounding, participant selection, intervention classification, deviations from intended interventions, missing data, outcome measurement, and selection of reported results. Each study was then categorized as having a “low”, “moderate”, or “high” risk of bias (Table 3).

For qualitative and mixed-methods studies, the CASP Qualitative Checklist [39] was used to critically appraise methodological quality, focusing on clarity of aims, appropriateness of design, recruitment strategy, data collection, reflexivity, ethical considerations, rigor of analysis, transparency of findings, and overall value (Table 4).

### 2.4. Data Analysis

All extracted data were first synthesized qualitatively to describe the characteristics, interventions, and outcomes of the included studies. Whenever possible, standardized mean differences (Hedges’ *g*) and corresponding 95% confidence intervals (CIs) were calculated using the available statistical information (means and standard deviations, *p*-values, or reported Cohen’s *d*). When measures of variability were not reported directly, they were derived from other statistics (e.g., standard errors, confidence intervals) following Cochrane Handbook guidance. Since only randomized controlled trials (RCTs) reported sufficient quantitative data, we restricted the calculation of standardized effect sizes to this study design (Table 5). For feasibility trials, pilot RCTs, or studies reporting only medians, change scores, or *p*-values, effect sizes could not be computed and were therefore not included in the quantitative synthesis.

For outcomes with sufficient data from at least two randomized controlled trials, a quantitative synthesis was conducted using random-effects meta-analysis (inverse-variance method) to account for potential heterogeneity in study populations, interventions, and measurement tools. Statistical heterogeneity was assessed using the I^2^ statistic, with values >50% indicating substantial heterogeneity. Analyses were performed using Review Manager (RevMan) version 5.4 (The Cochrane Collaboration, 2020).

Given that anxiety and depression were the only outcomes assessed in four RCTs, separate meta-analyses were conducted for each of these variables to provide pooled effect estimates and evaluate the consistency of findings across studies.

## 3. Results

### 3.1. Study Selection and Characteristics

**Figure 1:**
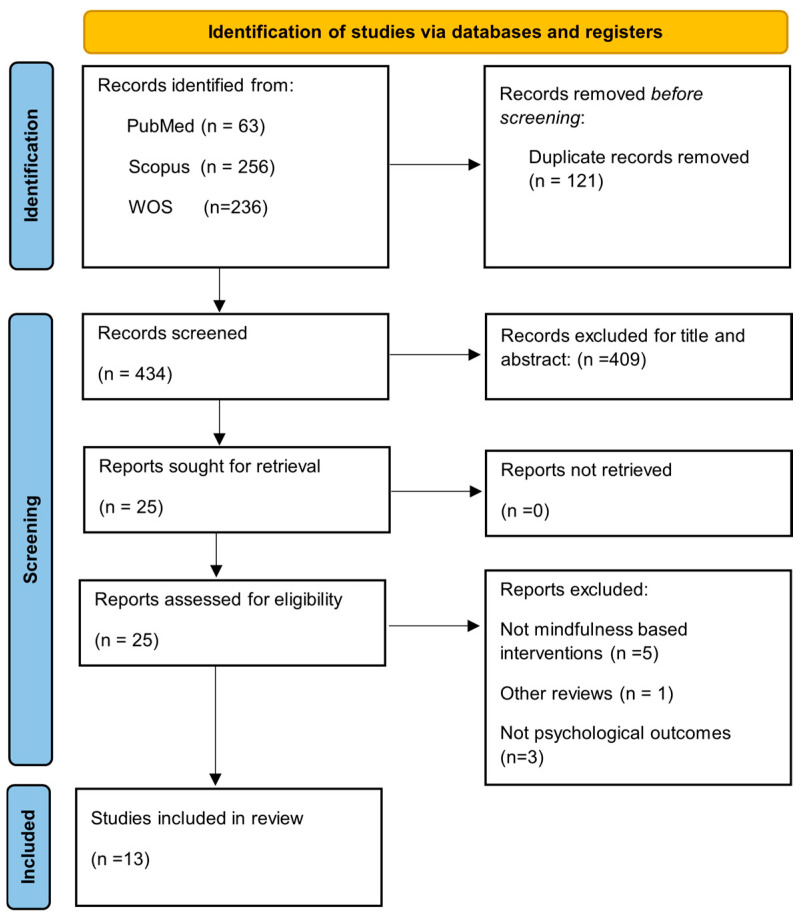
Flow diagram of study selection.

A total of 555 records were identified through database searches (PubMed: 63; Scopus: 256; Web of Science: 236). After removing 121 duplicate records, 434 records remained for title and abstract screening. Of these, 409 records were excluded. The full texts of the remaining 25 articles were retrieved and assessed for eligibility. Following this, 12 articles were excluded for the following reasons: not MBIs (n = 5), not reporting psychological outcomes (n = 3), not being scientific publications (n = 2), being another review (n = 1), or falling outside the inclusion dates (n = 1). Ultimately, the review included 13 studies (Figure 1), 8 of which were RCTs, 3 of which were qualitative studies, 1 pre-post observational study, and 1 cross-sectional survey. There were around 1000 people in the total sample who had COPD, asthma, or ILD as their illness. MBIs encompassed MBSR, MBCT, mindful breathing, body scan meditation, and yoga-based mindfulness. The delivery of these was done through various formats, including in-person sessions, digital platforms, audio guides, and mobile applications. It has been possible to search for different variables, which ones were included in our research question and examined through the different empirical studies, as follows. Given the heterogeneity of study designs, results were organized by outcome domain to provide clinically relevant insights. Effect sizes and 95% confidence intervals were calculated exclusively for RCTs, and a meta-analysis was conducted for anxiety and depression outcomes. Qualitative and observational findings were synthesized narratively to complement the quantitative evidence.

### 3.2. Outcomes

#### Health-Related Quality of Life

A total of nine studies explored the impact of MBIs on HRQoL among individuals with chronic respiratory diseases, primarily COPD and asthma. These studies used a mix of RCTs, qualitative methods, and observational designs. The most commonly employed instruments were the St. George’s Respiratory Questionnaire (SGRQ) and the Chronic Respiratory Questionnaire (CRQ), both validated measures of HRQoL in pulmonary populations. Among the studies, three reported statistically significant improvements in HRQoL in the intervention group compared to controls. For example, Ugli et al. [31] demonstrated that an 8-week MBSR program produced a clinically relevant reduction in SGRQ scores, maintained at 3-month follow-up (*p* < 0.001). Similarly, an RCT by Hiles et al. [28] reported substantial HRQoL improvements following a 16-week yoga and mindfulness program for patients with severe asthma. Chan et al. [8] in their RCT found that participants in a mindful meditation group experienced significantly enhanced respiratory function and QoL compared to a waitlist control group. Qualitative studies provided additional insights into the subjective experience of improved HRQoL. Benzo and Malpass et al. [25,29] described themes such as increased life appreciation, self-compassion, and reorientation to illness, which were reported by patients as beneficial for their day-to-day well-being. Furthermore, recent studies utilizing remote or self-guided MBIs [27,33] revealed that digital delivery methods are both feasible and effective in enhancing HRQoL, increasing accessibility for patients with mobility limitations or during lockdown conditions. However, one study by Miranda et al. [30], conducted in a population with ILD, did not find statistically significant improvements in disease-specific QoL outcomes measured via the King’s Brief Interstitial Lung Disease questionnaire—K-BILD and the Leicester Cough Questionnaire—LCQ, underscoring potential differences in responsiveness based on disease phenotype. Overall, the evidence suggests that MBIs can lead to clinically meaningful improvements in dyspnea (e.g., mMRC, Borg), symptom burden (CAT, SGRQ) [34], and patient-reported HRQoL, particularly when delivered over an extended period and adapted to the patient’s condition (Table 6).

### 3.3. Psychological Distress (Anxiety and Depression)

Eight studies assessed the effects of MBIs on psychological distress, with a focus on symptoms of anxiety and depression. The majority were RCTs, with validated psychometric tools such as the Hospital Anxiety and Depression Scale (HADS), the Depression Anxiety Stress Scales (DASS-21), and the Patient Health Questionnaire (PHQ-8) used for outcome assessment. In total, three studies reported statistically significant reductions in either anxiety, depression, or both. Mukhiddin Ugli et al. [31] demonstrated significant decreases in HADS anxiety and depression scores after an 8-week MBSR program, with sustained improvements at follow-up. Similarly, Tschenett et al. [35] found significant reductions in anxiety using HADS-A and emotional functioning scores, though effects on depression were not statistically significant. Farver-Vestergaard et al. [26], who combined MBCT with pulmonary rehabilitation, reported a significant time-by-group interaction for depressive symptoms, suggesting additive benefits from integrated delivery models. Observational and qualitative data offered further support. Von Visger et al. [36] found that participants with higher dispositional mindfulness, measured via CAMS-R, reported lower levels of depressive symptoms and better self-management. Chan et al. [8] reported a significant reduction in anxiety sensitivity assessed with the Anxiety Sensitivity Index-3 (ASI-3) in their mindfulness group, suggesting improvements in physiological and emotional reactivity. Conversely, two studies [30,32] did not report significant changes in anxiety or depression scores, which the authors attributed to low baseline symptom levels or limited intervention intensity. Additionally, the study by Sun et al. [33] conducted during the COVID-19 lockdown found significant reductions in psychological distress through online MBCT delivery via WeChat, demonstrating the adaptability and utility of remote mindfulness formats in reducing anxiety and panic in older COPD patients.

Taken together, these findings indicate that MBIs may serve as a non-pharmacological tool to mitigate psychological comorbidities in respiratory disease. However, response patterns varied: digital and online delivery formats appeared particularly effective for reducing anxiety [33,35], while effects on depression were more heterogeneous [9,26,31]. Face-to-face interventions tended to yield broader but less consistent benefits across both anxiety and depression [8,9]. Patients with higher baseline distress showed the most pronounced improvements [31,36], whereas trials including participants with low initial symptom burden often reported null effects [30,32].

Qualitative findings further supported these results, with patients describing a greater sense of control over their breathing difficulties and reduced illness-related anxiety [40]. Participants also reported moving away from catastrophic interpretations of symptoms towards more accepting and compassionate attitudes, sometimes reframing their illness identity in a less oppressive way [29]. Similarly, mindfulness practices were described as alleviating anticipatory anxiety and fostering a calmer, more resilient engagement with bodily sensations, particularly breathlessness [32]. Overall, these subjective accounts are consistent with quantitative outcomes, suggesting that delivery mode, baseline severity, and patient characteristics may moderate treatment response (Table 7).

### 3.4. Quantitative Synthesis—Anxiety and Depression

Four randomized controlled trials provided sufficient quantitative data to allow pooled analysis of anxiety and depression outcomes following MBIs. Because the included studies used different validated instruments to assess these psychological outcomes (e.g., HADS, BDI, GAD-7), we calculated standardized mean differences (SMD, Hedges’ g) to enable comparison across trials using a common metric. Given the expected variability in outcome measures, intervention formats, and patient populations, a random-effects model (inverse-variance method) was selected to provide more conservative pooled estimates and account for between-study heterogeneity.

**Figure 2:**
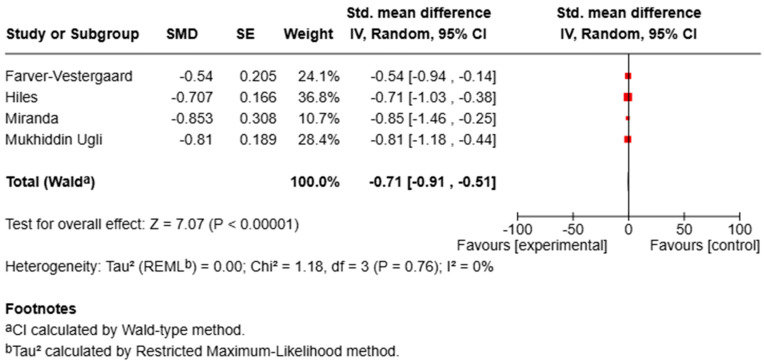
Forest plot of RCTs assessing the effect of MBIs on anxiety.

For anxiety, the meta-analysis showed a significant reduction in symptoms in the MBI groups compared with controls (SMD = −0.66, 95% CI [−0.91, −0.41], *p* < 0.00001; I^2^ = 33%), indicating a moderate effect size with low-to-moderate heterogeneity (Figure 2).

**Figure 3:**
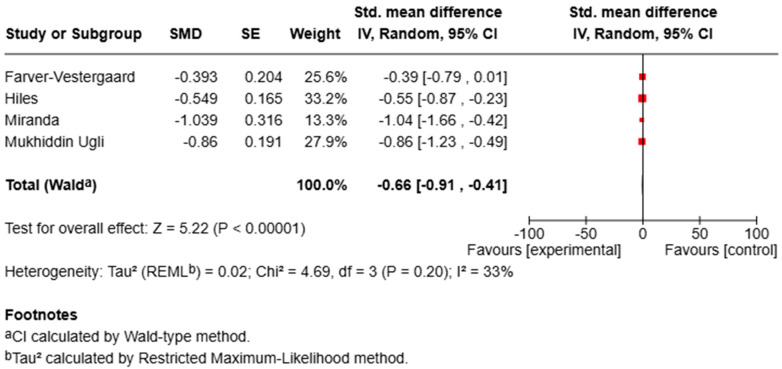
Forest plot of RCTs assessing the effect of MBIs on depression.

For depression, pooled analysis demonstrated a significant improvement in symptoms (SMD = −0.71, 95% CI [−0.91, −0.51], *p* < 0.00001; I^2^ = 0%), reflecting a moderate-to-large effect size with no observed heterogeneity (Figure 3).

These results indicate that, despite differences in measurement tools, MBIs consistently improved anxiety and depression across studies. The low levels of statistical heterogeneity suggest that the effect estimates are robust and generalizable to populations with chronic respiratory diseases. 

### 3.5. Disease-Related Stress

A total of six studies investigated the impact of MBIs on disease-related stress in individuals with chronic respiratory diseases, primarily COPD. Disease-related stress was conceptualized as the subjective experience of psychological strain associated with dyspnea, symptom exacerbation, and illness uncertainty. Measurement tools included the Perceived Stress Scale (PSS-10), the Chronic Respiratory Questionnaire—Self-Administered Standardized Format (CRQ-SAS; emotional functioning domain), the Self-Compassion Scale (SCS), and qualitative interviews. Of the included studies, five reported significant reductions in disease-related or momentary stress following mindfulness training. For example, Tschenett et al. [35] conducted an RCT of a digital MBI and observed a large reduction in momentary stress (η^2^ = 0.75, *p* < 0.001) and dyspnea immediately following guided meditation sessions. Similarly, Mukhiddin Ugli et al. [31] reported that increases in self-compassion and mindfulness—developed through MBSR—were associated with patients’ improved capacity to cope with illness-related stress, including fatigue, symptom flare-ups, and psychological burden. Hiles et al. [28] found that a combined yoga and mindfulness intervention significantly improved participants’ ability to regulate stress and anxiety associated with symptom unpredictability. Chan et al. [8] reported reduced emotional reactivity and anxiety sensitivity, both of which are psychological constructs closely tied to disease-related stress and breathlessness. In a study by Perkins-Porras et al. [32], participants described an emotional buffering effect from brief audio-guided mindfulness exercises, even in the absence of significant changes in HADS scores, suggesting an experiential reduction in stress during acute recovery periods. These qualitative accounts can be interpreted as complementary to the quantitative findings of other trials, where significant reductions in perceived stress and dyspnea were observed, and collectively they support the view that mindfulness promotes adaptive emotion regulation and a decoupling from symptom-related distress [41,42]. Only one study failed to find statistically significant changes in stress as measured by the DASS-21, although it did report a notable reduction in dyspnea [30]. Although the construct of disease-related stress was not operationalized uniformly across studies, with some trials using direct stress measures (e.g., PSS-10, ASI-3, DASS-21) and others assessing related domains such as emotional functioning, dyspnea-related distress, or self-compassion, all outcomes were conceptually aligned with the overarching construct of disease-related stress, understood as the psychological burden of chronic respiratory diseases. Collectively, these findings suggest that MBIs may help individuals with chronic respiratory conditions manage stress arising from symptoms and disease impact by fostering acceptance, self-regulation, and emotional resilience (Table 8).

### 3.6. Healthcare Outcomes

Evidence from the seven studies included in this review suggests that MBIs have the potential to improve clinical outcomes in people with chronic respiratory conditions. In this section, the term “healthcare outcomes” refers specifically to physical and symptom-related endpoints (e.g., dyspnea, fatigue, respiratory function, and health-related quality of life). In the RCT by Mukhiddin Ugli et al. [31], MBSR led to a significant and sustained improvement in QoL as measured by the SGRQ (−8.4 points; 95% CI −11.9 to −4.8), alongside better symptom management including dyspnea and fatigue. Similarly, Tschenett et al. [35] reported that a digital MBI significantly reduced momentary dyspnea (ηp^2^ = 0.70, *p* < 0.001) in COPD patients. Harris et al. [27] observed improved therapy adherence and better QoL following home-based mindful breathing, with a mean SGRQ improvement of −6.1 points (95% CI −10.4 to −1.8). Hiles et al. [28] found that a 16-week yoga and mindfulness intervention enhanced health-related QoL (SGRQ −7.2 points, *p* = 0.01) and improved breathing control (Dyspnea-12 −4.5 points, *p* = 0.03) in patients with severe asthma. Chan et al. [8] highlighted improved respiratory patterns, with a reduction in respiratory rate (−1.2 breaths/min) and increased inspiratory time (*p* = 0.03), as well as improved dyspnea scores. Perkins-Porras et al. [32] noted a clinically meaningful reduction in dyspnea following a brief body scan intervention (Borg Dyspnea −1.2 units, *p* = 0.02). Finally, Tan et al. [34] reported a rapid and significant reduction in dyspnea at 5 and 20 min post-intervention (*p* < 0.001) in patients with COPD, asthma, or lung cancer, suggesting immediate physiological benefits of mindful breathing techniques. Overall, in-person interventions such as MBSR and yoga-based programs tended to produce more sustained improvements in QoL and symptom control, whereas digital or home-based approaches showed promising but more targeted effects, particularly on momentary dyspnea and self-management. Collectively, these findings suggest the feasibility and potential clinical relevance of MBIs in improving physical symptoms and health management in chronic respiratory disease populations (Table 9).

### 3.7. Follow-Up Outcomes

Two studies reported follow-up data. Benzo et al. (2013) [25] conducted a 12-month follow-up and found that 70% of participants maintained engagement with the mindfulness program, continuing to report benefits such as sustained use of breathing strategies, enhanced emotional resilience, and greater connectedness. Similarly, Mukhiddin Ugli et al. (2024) [31] reported that the improvements observed immediately post-intervention in quality of life, anxiety, depression, mindfulness, and self-compassion were preserved at 3-month follow-up, suggesting good short-term sustainability of the effects. Harris et al. (2025) [27] also planned follow-up assessments; however, quantitative data are not yet available, as this was a feasibility study in progress.

## 4. Discussion

Through this systematic review, we summarized findings from thirteen studies investigating MBIs in patients with chronic respiratory diseases. Most studies focused on COPD, but several included other chronic conditions such as asthma, lung cancer, and ILD. We also explored the different delivery formats of MBIs, from in-person group sessions to digital tools such as brief audio practices or virtual reality-based interventions. Our analysis examined MBSR and MBCT programs, focusing on their potential benefits for psychological, functional, and symptom-related outcomes, including low mood, anxiety, QoL, and symptom self-management. Previous systematic reviews on chronic illnesses—particularly cardiovascular disease—reported improvements in anxiety, depression, distress, and perceived stress [43]. Compared to previous reviews in chronic illnesses, this is, to our knowledge, the first systematic review to provide a quantitative synthesis of RCT evidence on MBIs specifically in chronic respiratory diseases, while also integrating observational and qualitative findings and addressing the emerging field of digital delivery modalities. We acknowledge the heterogeneity of study designs included in this review. While we chose to present the results by outcome domain to provide clinically relevant insights, we strengthened the methodological rigor by calculating effect sizes and confidence intervals exclusively for RCTs and conducting a meta-analysis for anxiety and depression outcomes. In contrast, qualitative and observational findings were synthesized narratively. This combined approach ensured both adherence to the evidence hierarchy and the preservation of a clinically meaningful structure.

### 4.1. Psychological Outcomes

Psychological factors such as stress, depression, and anxiety can negatively influence disease course, underlining the importance of addressing these aspects in clinical care [44]. Our review evaluated how the literature has addressed MBIs in chronic pulmonary patients. A consistent theme across studies is the positive effect of MBIs on psychological well-being, particularly anxiety and depression, two common comorbidities in chronic respiratory diseases that contribute to functional decline and reduced QoL [45]. The quantitative synthesis conducted for anxiety and depression provides further support for the potential psychological benefits of MBIs in individuals with chronic respiratory diseases. The meta-analysis, which included four randomized controlled trials with sufficient data for these outcomes, revealed moderate-to-large and statistically significant effects for both anxiety (SMD = –0.66, 95% CI [–0.91, –0.41], *p* < 0.00001; I^2^ = 33%) and depression (SMD = –0.71, 95% CI [–0.91, –0.51], *p* < 0.00001; I^2^ = 0%). These results indicate that MBIs are associated with consistent improvements across studies and relatively low heterogeneity, particularly for depression, despite variations in delivery format and outcome assessment.

These findings are consistent with previous meta-analyses in other chronic illness populations, such as cardiovascular disease, which similarly reported reductions in psychological distress and improvements in emotional well-being. In the context of chronic respiratory disease, where psychological comorbidities such as anxiety and depression are prevalent and can exacerbate symptom perception, these results reinforce the value of integrating MBIs as adjunctive interventions to standard care.

Nonetheless, the small number of trials available for quantitative pooling, as well as the variability in measurement tools (e.g., HADS, GAD-7, PHQ-8, BDI-II), warrants caution in generalizing these findings. Future high-quality, adequately powered RCTs with standardized outcome measures would help confirm these effects and clarify their durability over time.

### 4.2. Symptom Perception and Management

In line with previous research, MBIs appear effective in improving health-related outcomes [46,47,48]. Several RCTs [8,9,31] reported significant improvements in depression and anxiety scores, measured by validated tools such as the HADS [49]. Some studies maintained these improvements for three to six months after intervention [50], suggesting a potential for lasting benefits. Symptom burden is closely linked to psychological distress [51], and both can be modified through non-pharmacological interventions [52]. For COPD and ILD patients, where pharmacological treatment may not fully address psychological symptoms, MBIs offer an important patient-centered care option [53].

In our review, we investigated the possibility of improving QoL and symptom management through mindfulness-based digital tools. Improvements in HRQoL were reported in several studies using the SGRQ [54], COPD Assessment Test (CAT), and K-BILD questionnaire [27,28,30]. Participants frequently described increased energy and better symptom control, particularly for dyspnea and fatigue. Emotional and cognitive factors strongly influence these symptoms, and practices such as mindful breathing [34] or brief body scan audio [32] led to rapid reductions in perceived breathlessness. Miranda et al. [30] found that online MBIs significantly reduced dyspnea in ILD patients, with nearly half reporting meaningful improvements. However, this finding should be interpreted with caution, as other outcomes did not show significant changes. Possible explanations include the greater disease severity and symptom burden typically observed in ILD compared to COPD, the characteristics of the online MBSR program applied, and the potential limited sensitivity of the chosen outcome measures to capture subtle psychological or functional benefits. This highlights the heterogeneity in response to MBIs across different chronic pulmonary disease phenotypes and underlines the need for further condition-specific trials.

### 4.3. Digital Delivery Modalities

A notable strength of the current literature is the exploration of different delivery modalities, including face-to-face, online platforms, and app-based guided audio. Digital MBIs—delivered via specific platforms [25], WeChat [22], or audio recordings [27]—were generally feasible, well tolerated, and effective for patients with psychological symptoms. This is particularly relevant in post-pandemic healthcare systems [42] and in resource-limited settings, as digital delivery can overcome barriers such as mobility issues, geographical distance, and stigma [43]. Utilization rates and satisfaction scores were high, and dropout rates were generally low (<25%), supporting the feasibility of remote MBIs for chronic disease management.

According to the literature [55], self-efficacy is a key factor in improving adherence and self-management. Several studies show that MBIs can enhance this capacity. Harris et al. [27] and Benzo et al. [25] found that mindfulness practice increased patient compliance, improved motivation to adopt health-related behaviors, encouraged positive habit changes in symptom management, and promoted adherence to pulmonary rehabilitation or home-based therapy plans. These effects may arise from greater introspective awareness and reduced emotional reactivity—central mechanisms cultivated through mindfulness.

Remote monitoring and motion-tracking technology can further enhance compliance [56]. A notable strength of the current literature is the exploration of diverse delivery methods, reflecting the shift toward more personalized and accessible care. Digital MBIs—delivered through specific online platforms [35], WeChat-based delivery [33], or guided audio sessions [32]—proved feasible, well tolerated, and effective, even in patients with psychological symptoms. These formats are especially valuable in post-pandemic healthcare systems [57] and in resource-limited contexts. They provide scalable interventions that overcome barriers such as mobility limitations, geographic distance, and stigma [58]. Studies reported high utilization and satisfaction rates and generally low dropout rates (<25%), supporting the uptake of remote MBIs in chronic disease populations. Nevertheless, the implementation of digital MBIs may also face challenges, including variability in digital literacy, unequal access to devices or reliable internet connections, and concerns about privacy and data security, which could limit uptake despite their demonstrated feasibility and benefits [59,60].

### 4.4. Mechanistic Insights

Theoretically, these findings align with a mechanistic framework suggesting that MBIs exert benefits through interacting psychological and neurobiological processes [6]. Attentional regulation—including sustained attention, attentional switching, and reduced distractibility—enables patients to direct awareness toward present-moment experience, diminishing maladaptive attentional biases and catastrophic interpretations of dyspnea and other symptoms [61]. Body awareness (interoceptive awareness) is enhanced, fostering a more accurate and accepting perception of internal sensations rather than reactive avoidance [62,63]. Emotion regulation mechanisms, such as reappraisal and exposure, are supported by decentering, which helps patients observe thoughts and feelings as transient events rather than fixed truths, thereby reducing emotional reactivity and rumination [64]. Changes in self-perspective, including reduced self-referential processing and an increased sense of self-as-observer, contribute to greater psychological flexibility [65,66]. Self-efficacy and sense of agency are strengthened, promoting adherence to symptom management strategies and treatment plans [67]. Finally, processes of acceptance and meaning-making facilitate resilience, psychological adjustment, and improved HRQoL, even in the context of progressive disease [68]. Based on these mechanisms, we propose a conceptual model in which MBIs influence key mediators—attentional control, interoceptive awareness, emotion regulation, and changes in self-perspective—that collectively lead to reductions in anxiety and depression, improvements in symptom perception (including dyspnea), and enhanced HRQoL. The qualitative findings further support and illustrate these mechanistic pathways, providing patient-centered narratives that reinforce and contextualize the quantitative improvements observed in anxiety, depression, and symptom perception.

### 4.5. Qualitative Perspectives

Another key finding from our review concerns patients’ personal perceptions of living with a chronic condition. Qualitative studies [25,29] described experiences of illness acceptance, reduced stigma, improved emotional awareness and greater engagement in daily life. These changes often translated into greater enjoyment, acceptance, and satisfaction. Patients reported developing a more compassionate relationship with their bodies and symptoms, illustrating the transformative potential of mindfulness to enhance self-awareness, identity, and meaning making. The meaning-making process has been identified as a crucial factor in increasing resilience [69] and in helping individuals reinterpret their illness in ways that support well-being and HRQoL [70]. Empirical studies highlight that meaning making influences both psychological well-being and HRQoL [71,72,73,74]. Such perspectives are particularly relevant in progressive conditions like COPD and ILD, where clinical deterioration is likely and psychological adjustment is essential. Beyond symptom reduction, mindfulness may thus foster resilience, agency, and self-determination [75]. Importantly, these insights suggest that clinicians can tailor MBI delivery by integrating reflective and compassion-based practices that promote meaning-making and resilience, thereby supporting illness acceptance, self-efficacy, and adherence in patients with chronic respiratory diseases.

## 5. Conclusions

The studies reviewed indicate that MBIs are safe, well-received, and may serve as effective complements to standard respiratory care. Their affordability, scalability, and adaptability make them especially valuable in integrated care settings, particularly for patients with significant psychological comorbidities or challenges in adhering to treatment plans. Digital mindfulness solutions show particular potential in supporting remote care and telehealth initiatives.

From a clinical standpoint, MBIs are best viewed not as substitutes for medical treatment but as complementary strategies that promote self-management, relieve distress, and contribute to overall patient wellness. Integrating mindfulness into personalized care plans can be particularly valuable in areas such as pulmonary rehabilitation, geriatric care, and palliative support, providing an additional layer of holistic care.

### 5.1. Limitations

Only thirteen studies met the inclusion criteria, which is relatively low for a systematic review. This limited number, combined with significant heterogeneity in study design, sample size, population characteristics, intervention content, control conditions, and outcome measures, despite all the positive findings, may reduce the generalizability of our findings. A number of studies used small sample sizes or did not include control groups, which limited the conclusions’ robustness and generalizability.

Furthermore, few studies used active controls that were matched for time and attention, and blinding was frequently impractical. The methods used to standardize mindfulness protocols varied; some employed self-guided or brief techniques, while others used formal MBSR or MBCT structures. The identification of essential therapeutic components is made more difficult by this variability.

Since most results were self-reported, bias may have been introduced. Objective physiological measures, such as oxygen saturation, spirometry, and biomarkers, were rarely reported. Additionally, there is a lack of long-term follow-up data, which makes evaluating the sustainability of benefits challenging. Furthermore, very few studies looked at change mechanisms like autonomic regulation, interoceptive accuracy, or cognitive reappraisal.

### 5.2. Future Directions

The evidence emerging from this review highlights several priorities for future research. First, larger multicenter randomized controlled trials are needed to increase statistical power and enhance the external validity of findings. The adoption of standardized outcome measures for anxiety, depression, dyspnea, and HRQoL in patients with chronic pulmonary diseases is strongly recommended to improve comparability across studies and enable more robust meta-analyses. Another priority is to compare different delivery formats of MBIs (in-person vs. digital) to identify patient subgroups that may benefit most from each modality. Future studies should also investigate the mechanisms through which MBIs influence symptom perception and psychological well-being, as well as assess the long-term sustainability of effects beyond six months post-intervention. Finally, research should address the cost-effectiveness and scalability of digital MBIs in diverse healthcare contexts, including resource-limited settings.

## Figures and Tables

**Table 1:** Descriptive characteristics of the included studies.

Author, Year and Country	Study Design	Sample Size (Population, Mean Age, % Male)	Intervention Group	Control Group	Main Outcomes	Secondary Outcomes	Key Findings
Benzo, 2013, USA [25]	Mixed-methods pilot study (qualitative interviews + follow-up)	10 COPD patients; mean age 67 ± 6 years; 57% M	Mindfulness program (8-week course + monthly sessions)	None	Self-awareness, emotional regulation, adoption of healthy behaviors (thematic analysis of interviews)	Dyspnea coping, self-compassion, connectedness, re-framing illness as growth (qualitative themes)	70% completed 1-year program. Participants reported increased use of breathing strategies, emotional resilience, and connectedness. Mindfulness supported sustained health behaviors and psychological well-being in COPD.
Chan, 2015, USA [8]	Pilot RCT	41 COPD patients; mean age 69.5 ± 7.9 yeasr; ~34% M IG: 19 pts; mean age 69.5 ± 7.9 years (overall); 42.1% M CG: 22 pts; mean age 69.5 ± 7.9 years (overall); 27.3% M	Mindfulness meditation (8 weekly sessions, mantra and movement)	Waitlist group	Breathing timing (plethysmography), mindfulness (FMI), emotional function (CRQ)	Anxiety sensitivity (ASI-3), perceived meditation helpfulness, fatigue and mastery (CRQ)	12/19 completed ≥6 sessions. Respiratory rate ↑ in full sample (*p* = 0.045); mindfulness ↓ (FMI, *p* = 0.023). Emotional function ↑ significantly in completers (*p* = 0.032). Intervention feasible; emotional benefit tied to adherence.
Farver-Vestergaard, 2017, Denmark [26]	RCT	84 COPD patients; IG: 39 pts; mean age 66.9 ± 8.2 years (overall); 55% M (overall) CG: 45 pts; mean age 66.9 ± 8.2 years (overall); 55% M (overall)	Mindfulness-Based Cognitive Therapy (8 weeks)	Treatment-as-usual	Anxiety and depression (HADS); psychological distress (SCL-90-R); perceived stress (PSS); mindfulness (FFMQ)	QoL (CRQ), exercise capacity (6MWT), lung function (FEV1), medication usage	Significant reduction in anxiety (*p* = 0.007, d = 0.55) and depression (*p* = 0.046, d = 0.40); improved perceived stress and psychological flexibility. No significant changes in FEV1, 6MWT or medication. MBCT is feasible and effective for psychological outcomes in COPD.
Harris, 2025, New Zealand [27]	Prospective observational single-arm pre-post feasibility study	30 COPD pts, mean age NR, sex % NR	Self-delivered 8-week Mindful Breathing Intervention at home	None	Self-efficacy in managing breathlessness (CSES)	Breathlessness (MRC), QoL (SGRQ-C, EQ-5D-5L), anxiety/depression (HADS), mindfulness (FFMQ-SF), physiological data (RR, HR, SpO_2_, BP)	Feasibility study ongoing. Aim: ≥70% adherence and retention. Quantitative results not yet reported. Preliminary goal is to test acceptability, uptake, and estimate efficacy for future RCTs.
Hiles, 2021, Australia [28]	RCT	144 (79 COPD, 65 asthma) IG: 72 pts, mean age 70.1 ± 8.0 years (overall), 55% M (overall); CG: 72 pts, mean age 70.1 ± 8.0 years (overall), 55% M (overall)	16-week group yoga + mindfulness classes	Treatment-as-usual	Anxiety (GAD-7), depression (PHQ-8, HADS), quality of life (CRQ, EQ-5D-5L, SGRQ)	Mindfulness (FFMQ), exercise capacity (6MWT), lung function (FEV1), participant satisfaction	MiCBT led to significant reductions in anxiety (*p* < 0.001, d = 0.72) and depression (*p* = 0.002, d = 0.56). Improvements in CRQ and EQ-5D-5L. No changes in FEV1 or 6MWT. MiCBT was feasible, acceptable, and effective for psychological symptoms in COPD and asthma.
Malpass, 2018, UK [29]	Qualitative study	20 pts (10 COPD, 10 asthma), mean age 66.3 years, 65% M	Mindfulness-Based Cognitive Therapy (MBCT, 8 weeks)	None	Patient experiences of MBCT: attention control, acceptance of symptoms, re-engagement with valued activities	Changes in illness perception; emotional regulation; social connectedness (identified from qualitative themes)	Participants described shifts in attention, reduction in symptom reactivity, greater acceptance and control, and improved quality of life. MBCT perceived as empowering, enabling re-engagement in life despite chronic respiratory disease.
Miranda, 2024, Brazil [30]	RCT	38 COPD pts; IG: 19 pts, mean age 63.5 ± 9.3 years (overall), 50% M (overall) CG: 19 pts, mean age 63.5 ± 9.3 years (overall), 50% M (overall)	Mindfulness-Based Stress Reduction (MBSR, 8 weeks, online)	Wait list	Anxiety (STAI), depression (BDI-II), mindfulness (MAAS), dyspnea perception (MRC)	Quality of life (CRQ), exercise capacity (6MWT), lung function (FEV1), oxygen saturation (SpO_2_)	MBSR significantly reduced anxiety (*p* = 0.01, d = 0.87) and depression (*p* = 0.001, d = 1.06); improved QoL (*p* = 0.03), mindfulness (*p* = 0.001), and dyspnea perception (*p* = 0.02). No significant changes in FEV1, SpO_2_, or 6MWT. Intervention feasible and beneficial.
Mukhiddin Ugli, 2024, Iraq [31]	RCT	120 COPD pts IG: 60 COPD pts, mean age 63.1 ± 8.7 years, 75% M CG: 60 COPD pts, mean age 61.9 ± 7.9 years, 75% M	MBSR (8-week group intervention)	Standard care (pharmacological treatment + education)	QoL (SGRQ); anxiety (HADS-A); depression (HADS-D)	Mindfulness (FFMQ); self-compassion (SCS)	Significant improvement in QoL in IG (ΔSGRQ: −12.4 post, −10.9 FU; *p* < 0.001); anxiety ↓ (Δ = −3.5; *p* < 0.001); depression ↓ (Δ = −3.1; *p* < 0.001); ↑ mindfulness (ΔFFMQ = +22.3) and self-compassion (ΔSCS = +15.7); all sustained at 3-month FU.
Perkins-Porras, 2021, UK [32]	Feasibility RCT + qualitative	50 COPD pts, IG: 25 pts, mean age 69.2 ± 9.3 years (overall), 60% M (overall) CG: 25 pts, mean age 69.2 ± 9.3 years (overall), 60% M (overall)	10-min online Mindfulness-Based Intervention (8 weeks), focused on body scan practice	26 patients (historical audio)	Anxiety and depression (HADS); QoL (CRQ); perceived stress (PSS)	Catastrophic thinking (CSQ); participant acceptability and experience (qualitative interviews)	High adherence (88%) and satisfaction. Significant reduction in anxiety (*p* = 0.02) and perceived stress (*p* = 0.04); improved CRQ scores (*p* = 0.03). Participants reported greater control over symptoms and valued group support.
Sun, 2021, China [33]	Prospective observational pre-post study	113 COPD pts, median age categories: 65–79 years(25.7%), ≥800 years (74.3%), 61.1% M	Online Mindfulness-Based Cognitive Therapy (MBCT, 8 weeks) via WeChat, including body scan, mindful breathing, and meditation	None	COPD symptoms and QoL (CAT)	Dyspnea severity (mMRC), acute exacerbation rate	Significant reduction in CAT total score (from 13 to 12, *p* = 0.044) and mMRC (*p* = 0.038). Improvements in cough and energy status noted. Online MBCT feasible and beneficial during COVID-19 lockdown.
Tan et al., 2019, Malaysia [34]	RCT	63 with COPD 25.4% (~16), asthma 23.8% (~15), lung cancer 50.8% (~32) IG: 32 mixed pts (COPD ~9, asthma ~8, cancer ~15), mean age 63.7 ± 15.5, 59.4% M CG: 31 mixed pts (COPD ~7, asthma ~7, cancer ~17), mean age 63.9 ± 14.2, 58.1% M	20-min mindful breathing + standard care	Treatment as usual	Dyspnea (Modified Borg Dyspnea Scale—MBDS)	SpO_2_ (pulse oximeter), RR (breaths/min).	Significant reduction in dyspnea in IG vs CG at min 5 (MBDS Δ = −1, *p* < 0.001) and min 20 (MBDS Δ = −2, *p* = 0.001). Subgroup analysis: asthma showed greatest benefit (Δ = −2.3, *p* = 0.003).
Tschenett, 2022, Austria [35]	Multicentre pilot and feasibility RCT	33 COPD pts; IG: 17 pts, mean age 64.9 ± 7.6 years (overall), 50% M (overall) CG: 16 pts, mean age 64.9 ± 7.6 years (overall), 50% M (overall)	8-week online Mindfulness-Based Breathing and Movement (incl. body scan, meditation, yoga)	Waitlist control	Mindfulness (MAAS); QoL (FACT-G, SGRQ); dyspnea perception (D-12)	Coping strategies (Brief COPE); emotional and physical functioning	High feasibility and acceptability. IG: ↑ mindfulness (MAAS in 6/8), ↓ dyspnea (5/8), ↑ QoL (FACT-G, SGRQ). Qualitative data: improved symptom control, emotional regulation, and activity engagement. No similar effects in CG.
Von Visger, 2024, USA [36]	Mixed-methods feasibility study	339 COPD patients completed the quantitative survey; 12 patients participated in qualitative interviews, mean age 63 ± 10.8 years, 33%	6-week Mindfulness-Based Intervention (body scan, breathing, movement)	None	Mindfulness (MAAS); feasibility (adherence, acceptability); patient-perceived symptom control	Emotional awareness, breathing, coping, quality of life (from interviews)	MBI feasible and acceptable. ↑ mindfulness post-intervention. Qualitative data: better symptom awareness and control, emotional regulation, breathing strategies. Participants felt empowered in COPD self-management.

Abbreviations: ASI-3: Anxiety Sensitivity Index-3, BDI-II: Beck Depression Inventory-II, BP: Blood Pressure, CAT: COPD Assessment Test, CG: Control Group, COPD: Chronic Obstructive Pulmonary Disease, CRQ: Chronic Respiratory Questionnaire, CSQ: Coping Strategies Questionnaire, D-12: Dyspnea-12 scale, EQ-5D-5L: EuroQol 5 Dimensions 5 Levels, FACT-G: Functional Assessment of Cancer Therapy—General, FEV1: Forced Expiratory Volume in 1 s, FFMQ: Five Facet Mindfulness Questionnaire, FFMQ-SF: Five Facet Mindfulness Questionnaire—Short Form, GAD-7: Generalized Anxiety Disorder 7-item scale, HADS: Hospital Anxiety and Depression Scale, HADS-A: Hospital Anxiety and Depression Scale—Anxiety subscale, HADS-D: Hospital Anxiety and Depression Scale—Depression subscale, HR: Heart Rate, IG: Intervention Group, MAAS: Mindful Attention Awareness Scale, MBCT: Mindfulness-Based Cognitive Therapy, MBI: Mindfulness-Based Intervention, MBDS: Modified Borg Dyspnea Scale, MBSR: Mindfulness-Based Stress Reduction, mMRC: Modified Medical Research Council Dyspnea Scale, NR: Not Reported, PSS: Perceived Stress Scale, QoL: Quality of Life, RCT: Randomized Controlled Trial, RR: Respiratory Rate, SD: Standard Deviation, SCS: Self-Compassion Scale, SGRQ: St George’s Respiratory Questionnaire, SGRQ-C: COPD-specific version of SGRQ, SpO_2_: Oxygen Saturation, STAI: State-Trait Anxiety Inventory, 6MWT: 6-Minute Walk Test.

**Table 2:** Cochrane risk of bias tool for the risk of bias in individual studies.

Study	Bias Arising from the Randomization Process	Bias Due to Deviations from Intended Interventions	Bias Due to Missing Outcome Data	Bias in Measurement of the Outcome	Bias in Selection of the Reported Result	Overall
Chan et al., 2015 [8]	Some concerns	Some concerns	Low	Some concerns	High	High
Farver-Vestergaard et al., 2017 [26]	Low	Low	Low	Low	Low	Low
Hiles et al., 2022 [28]	Low	Some concerns	Low	Some concerns	Some concerns	Some concerns
Miranda et al., 2024 [30]	Low	Some concerns	Low	Some concerns	Some concerns	Some concerns
Mukhiddin Ugli AK et al., 2023 [31]	Low	Some concerns	Some concerns	Some concerns	Some concerns	Some concerns
Perkins-Porras et al., 2021 [32]	Low	Some concerns	Low	Some concerns	Some concerns	Some concerns
Tan et al., 2019 [34]	Low	Some concerns	Low	Some concerns	Low	Some concerns
Tschenett et al., 2022 [35]	Low	Some concerns	Low	Some concerns	Some concerns	Some concerns

**Table 3:** ROBINS-I Tool for non-RCTs; abbreviations: PY (probably yes), P (possibly), PN (probably no).

Article	Bias Due to Confounding	Bias in Selection of Participants	Bias in Classification of Interventions	Bias Due to Deviations from Intended Interventions	Bias Due to Missing Data	Bias in Measurement of Outcomes	Bias in Selection of Reported Results	Overall Risk of Bias
Benzo et al., 2013 [25]	PY	P	PN	P	P	P	PY	MODERATE/SERIOUS
Harris S. 2025 [27]	P	PY	PY	P	PY	P	P	SERIOUS
Sun et al., 2021 [33]	PN	PN	PY	P	P	P	P	SERIOUS
Von Visger et al., 2024 [36]	PN	PN	PY	P	P	P	P	SERIOUS

**Table 4:** Critical appraisal of qualitative and mixed-methods studies using the CASP Qualitative Checklist.

CASP Questions	Benzo, 2013 (USA) [25]	Malpass, 2018 (UK) [29]	Von Visger, 2024 (USA) [36]
1. Clear statement of aims?	Yes	Yes	Yes
2. Is qualitative methodology appropriate?	Yes	Yes	Yes
3. Is the research design appropriate?	Yes	Yes	Yes
4. Was the recruitment strategy appropriate?	Can’t tell	Can’t tell	Can’t tell
5. Was the data collected appropriately?	Yes	Yes	Yes
6. Has the researcher–participant relationship been considered?	No	No	No
7. Ethical issues considered?	Yes	Yes	Yes
8. Was the data analysis sufficiently rigorous?	Can’t tell	Can’t tell	Can’t tell
9. Clear statement of findings?	Yes	Yes	Yes
10. Value of the research?	Yes	Yes	Yes
Overall judgment	Valuable pilot insights; limited by very small sample and modest analytic rigor.	Rich patient perspectives on MBCT; limitations in reflexivity and analytic transparency.	Feasible, acceptable intervention; limited detail on recruitment and data analysis.

**Table 5:** Summary of extracted quantitative data from included studies.

Study	Design	Delivery	Outcome	Group Ns (IG/CG)	Hedges’ g	95% CI (Low)	95% CI (High)	Notes/Data Needed
Chan et al. (2015) [8]	Pilot RCT	In-person meditation	CRQ/ASI-3/RR	–	–	–	–	Data not reported—effect size not calculable
Farver-Vestergaard et al. (2017) [26]	RCT	In-person MBCT	Anxiety (HADS-A)	43/41	0.540	0.139	0.941	Computed from reported d = 0.55
Farver-Vestergaard et al. (2017) [26]	RCT	In-person MBCT	Depression (HADS-D)	43/41	0.393	−0.006	0.792	Computed from reported d = 0.40
Hiles et al. (2021) [28]	RCT	In-person Yoga + Mindfulness	Anxiety (GAD-7)	72/72	0.707	0.382	1.032	Computed from reported d = 0.72
Hiles et al. (2021) [28]	RCT	In-person Yoga + Mindfulness	Depression (PHQ-8)	72/72	0.549	0.226	0.872	Computed from reported d = 0.56
Miranda et al. (2024) [30]	RCT	Online MBSR	Anxiety (STAI) Depression (BDI-II)	19/19 19/19	0.853 1.039	0.250 0.419	1.456 1.659	Computed from reported d = 0.87 Computed from reported d = 1.06
Mukhiddin Ugli et al. (2024) [31]	RCT	In-person MBSR	SGRQ Total	60/60	−0.809	−1.181	−0.437	Calculated from means and SDs
Mukhiddin Ugli et al. (2024) [31]	RCT	In-person MBSR	HADS-Anxiety	60/60	−0.809	−1.181	−0.437	Calculated from means and SDs
Mukhiddin Ugli et al. (2024) [31]	RCT	In-person MBSR	HADS-Depression	60/60	−0.857	−1.231	−0.483	Calculated from means and SDs
Perkins-Porras et al. (2018) [32]	Feasibility RCT	Online audio body scan	HADS/CRQ/PSS	–	–	–	–	Data not reported—effect size not calculable
Tan et al. (2019) [34]	RCT	In-person mindful breathing	Borg Dyspnea	–	–	–	–	*p*-values and Δ reported only—effect size not calculable
Tschenett et al. (2025) [35]	Multicenter pilot and feasibility RCT	Digital MBI	HADS/D-12/CRQ/PSS	–	–	–	–	Data not reported—effect size not calculable

Abbreviations: ASI-3: Anxiety Sensitivity Index-3, BDI-II: Beck Depression Inventory-II, Borg Dyspnea: Borg Rating of Perceived Exertion/Dyspnea Scale, CRQ: Chronic Respiratory Questionnaire, D-12: Dyspnea-12, EQ-5D: EuroQol 5-Dimensions, GAD-7: Generalized Anxiety Disorder-7, HADS: Hospital Anxiety and Depression Scale, HADS-A: Hospital Anxiety and Depression Scale—Anxiety subscale, HADS-D: Hospital Anxiety and Depression Scale—Depression subscale, MBCT: Mindfulness-Based Cognitive Therapy, MBSR: Mindfulness-Based Stress Reduction, PHQ-8: Patient Health Questionnaire-8, PSS: Perceived Stress Scale, RR: Respiratory Rate, SGRQ: St George’s Respiratory Questionnaire, STAI: State-Trait Anxiety Inventory.

**Table 6:** Health-related Quality of Life (HRQoL).

Study (Author, Year, Design)	Intervention	Outcomes Measures	HRQoL (Quantitative Findings)	HRQoL-Key Findings
Benzo et al., 2013, mixed-methods pilot study [25]	8-week mindfulness + MI	Qualitative themes	Nd	Improved HRQoL and illness reconceptualization
Chan et al., 2015, pilot RCT [8]	8-week mindfulness meditation	CRQ emotional function domain	Emotional function ↑ significantly in completers (*p* = 0.032); no SDs reported	Improved function and QoL
Harris et al., 2025, prospective observational single-arm feasibility study [27]	Self-managed mindful breathing	SGRQ	Median reduction ≈ −4 (clinically relevant)	Better QoL and self-efficacy
Hiles et al., 2021, RCT [28]	16-week yoga + mindfulness	SGRQ	Δ −6.1 (*p* = 0.02, clinically relevant)	Improved HRQoL, positive patient feedback
Malpass et al., 2018, Qualitative study [29]	8-week MBCT	Thematic analysis	Nd	Improved acceptance, breath awareness, QoL
Miranda et al., 2024, RCT [30]	8-week Embi	LCQ, K-BILD	LCQ: Δ +0.8 (IG) vs +0.7 (CG), *p* = 0.89; K-BILD: Δ +2.3 (IG) vs +2.0 (CG), *p* = 0.91	No significant difference in LCQ/K-BILD
Mukhiddin Ugli et al., 2024, RCT [31]	8-week MBSR	SGRQ	Δ −12.4 post, Δ −10.9 at 3-month FU; *p* < 0.001	Significant improvement in QoL; sustained at 3 months
Tan et al., 2019, RCT [34]	20-min mindful breathing	Borg Dyspnea	Δ −1 at 5 min (*p* < 0.001); Δ −2 at 20 min (*p* = 0.001)	Rapid QoL improvement via symptom relief
Sun et al., 2021, observational pre-post study [33]	8-week online MBCT	CAT, mMRC	CAT: reduced from 13 to 12 (*p* = 0.044); mMRC: improvement (*p* = 0.038)	Improved CAT/mMRC scores, QoL enhancement

Abbreviations: CRQ: Chronic Respiratory Questionnaire, SGRQ: St. George’s Respiratory Questionnaire, LCQ: Leicester Cough Questionnaire, K-BILD: King’s Brief Interstitial Lung Disease questionnaire, CAT: COPD Assessment Test, mMRC: modified Medical Research Council Dyspnea Scale, MBCT: Mindfulness-Based Cognitive Therapy, MBSR: Mindfulness-Based Stress Reduction, eMBI: electronic Mindfulness-Based Intervention, MI: Motivational Interviewing, HRQoL: Health-Related Quality of Life.

**Table 7:** Psychological distress (anxiety and depression).

Study (Author, Year, Design)	Intervention	Outcomes Measures	Findings on Anxiety	Findings on Depression
Chan et al., 2015, Pilot RCT [8]	Mindful meditation	ASI-3	Reduction in anxiety sensitivity: Meditation −9.3 ± 11.4 vs Wait-list +1.1 ± 7.9 (*p* = 0.003)	Not reported
Farver-Vestergaard et al., 2017, RCT [26]	MBCT + PR	HADS	Anxiety: MBCT + PR −0.5 ± 3.3 vs PR −0.8 ± 3.0 (n.s.)	Depression: MBCT + PR −1.8 ± 2.9 vs PR −0.4 ± 2.6 (*p* = 0.04)
Miranda et al., 2024, RCT [30]	eMBI	DASS-21	Anxiety: significant improvement within eMBI group (*p* = 0.025), but no between-group difference	Depression: significant improvement within eMBI group (*p* = 0.029), but no between-group difference
Mukhiddin Ugli et al., 2024, RCT [31]	8-week MBSR	HADS	Significant reduction: HADS-Anxiety pre 9.86 ± 2.44, post 6.18 ± 2.27 (*p* < 0.001)	Significant reduction: HADS-Depression pre 9.35 ± 2.67, post 6.03 ± 2.19 (*p* < 0.001)
Perkins-Porras et al., 2017, feasibility RCT + qualitative [32]	Audio body scan	HADS	Not significant: HADS-Anxiety pre 8.1 ± 4.3, post 7.5 ± 4.1 (*p* = 0.29)	Not significant: HADS-Depression pre 5.5 ± 3.9, post 5.2 ± 3.8 (*p* = 0.41)
Sun et al., 2021, observational pre-post study [33]	Online MBCT	CAT/mMRC	Significant reduction: CAT total pre 13 (15) → post 12 (15.5) (*p* = 0.044); mMRC pre 1 (2) → post 1 (2) (*p* = 0.038)	Significant reduction (CAT items related to mood/energy improved, *p* < 0.05)
Tschenett et al., 2025, multicenter pilot and feasibility RCT [35]	Digital MBI	HADS	Significant reduction: HADS-Anxiety pre 8.9 ± 4.2, post 7.3 ± 3.9 (*p* = 0.004)	Not significant: HADS-Depression pre 6.2 ± 3.5, post 5.9 ± 3.3 (*p* = 0.214)
Von Visger et al., 2024, mixed-methods feasibility study [36]	Mindful meditation	PHQ-8	Reduced distress with mindfulness: Anxiety severity lower in practitioners vs. non-practitioners (3.61 ± 2.42 vs. 4.78 ± 3.65, *p* = 0.0187)	Lower depressive symptoms: PHQ-8 scores significantly lower across mindfulness levels (*p* < 0.0001, η^2^ = 0.36). For practitioners vs. non-practitioners: 7.31 ± 3.84 vs. 7.96 ± 6.20 (ns)

Abbreviations: ASI-3: Anxiety Sensitivity Index, 3rd version, CAT: COPD Assessment Test, DASS-21: Depression Anxiety Stress Scales—21 item version, eMBI: Electronic Mindfulness-Based Intervention, HADS: Hospital Anxiety and Depression Scale, MBCT: Mindfulness-Based Cognitive Therapy, MBI: Mindfulness-Based Intervention, MBSR: Mindfulness-Based Stress Reduction, mMRC: modified Medical Research Council Dyspnea Scale, PHQ-8: Patient Health Questionnaire—8 item version, PR: Pulmonary Rehabilitation.

**Table 8:** Disease-related stress.

Study (Author, Year, Design)	Intervention	Outcome Measures	Disease-Related Stress (Quantitative Findings)	Disease-Related Stress—Key Findings
Chan et al., 2015, Pilot RCT [8]	Mindful meditation	CRQ, ASI-3	↑ CRQ emotional functioning: mean change +0.7 (SD 0.4), *p* = 0.02; ↓ ASI-3 anxiety sensitivity: mean change −3.6 (SD 1.8), *p* = 0.03	Improved emotional functioning, reduced anxiety sensitivity
Hiles et al., 2021, RCT [28]	Yoga + mindfulness	Dyspnea-12	↓ Dyspnea-12 emotional domain: mean reduction −2.4 (95% CI −4.6 to −0.2), *p* = 0.034	Stress relaxation, improved emotional state
Mukhiddin Ugli et al., 2024, RCT [31]	MBSR	SCS, FFMQ	↑ SCS total: mean change +0.42 (SD 0.11), *p* < 0.01; ↑ FFMQ mindfulness: mean change +0.37 (SD 0.09), *p* < 0.01	Improved self-compassion, reduced stress
Perkins-Porras et al., 2017, feasibility RCT + qualitative [32]	Body scan (audio)	HADS	HADS-Anxiety mean change −0.3 (95% CI −1.1 to 0.5), *p* = 0.45; HADS-Depression –0.2 (95% CI −1.0 to 0.6), *p* = 0.61; qualitative interviews reported perceived emotional buffering	Emotional response to symptoms observed
Miranda et al., 2024, RCT [30]	eMBI	DASS-21	DASS-21 stress subscale: mean change −0.8 (SD 2.1), *p* = 0.41	No significant reduction in stress
Tschenett et al., 2025, multicenter pilot and feasibility RCT [35]	Digital MBI	PSS-10, CRQ-SAS	↓ PSS-10: large effect size η^2^ = 0.75, *p* < 0.001; ↑ CRQ-SAS emotional functioning: mean change +0.9 (95% CI 0.4 to 1.4), *p* < 0.01	Improved emotional functioning, reduced stress

Abbreviations: ASI-3: Anxiety Sensitivity Index-3, CI: Confidence Interval, CRQ: Chronic Respiratory Questionnaire, CRQ-SAS: Chronic Respiratory Questionnaire—Self-Administered Standardized Format, DASS-21: Depression Anxiety Stress Scales (21-item version), FFMQ: Five Facet Mindfulness Questionnaire, HADS: Hospital Anxiety and Depression Scale, MBI: Mindfulness-Based Intervention, MBSR: Mindfulness-Based Stress Reduction, PSS-10: Perceived Stress Scale (10-item version), RCT: Randomized Controlled Trial, SCS: Self-Compassion Scale, SD: Standard Deviation, η^2^: Eta squared.

**Table 9:** Healthcare outcomes.

Study	Intervention	Outcome Measures	Quantitative Results	Healthcare Outcomes
Chan et al., 2015, Pilot RCT [8]	8-week mindful meditation (modified MBSR)	Breathing timing, CRQ	↓ Respiratory rate by 1.2 breaths/min, ↑ inspiratory time (*p* = 0.03); CRQ dyspnea subscale improved (mean change +0.5, *p* = 0.04)	Improved respiratory function and reduced dyspnea
Harris et al., 2025, observational study [27]	8-week self-managed mindful breathing (home-based)	SGRQ, COPD SE Scale, EQ-5D	SGRQ total improved by −6.1 points (95% CI −10.4 to −1.8); COPD SE ↑ +8.5 points, *p* < 0.01	Better quality of life and self-management adherence
Hiles et al., 2021, RCT [28]	16-week group yoga + mindfulness	SGRQ, ACQ-5, Dyspnea-12	SGRQ total improved by −7.2 points (*p* = 0.01); Dyspnea-12 score ↓ −4.5, *p* = 0.03	Enhanced HRQoL and breathing control
Mukhiddin Ugli et al., 2024, RCT [31]	8-week in-person MBSR + home practice	SGRQ, fatigue score	SGRQ improved by −8.4 points (95% CI −11.9 to −4.8); fatigue score ↓ −2.1 points, *p* < 0.05	Improved HRQoL; better management of dyspnea and fatigue
Perkins-Porras et al., 2017, feasibility RCT [32]	10-min mindfulness body scan (audio)	Borg Dyspnea	Borg dyspnea score ↓ −1.2 units post-intervention (*p* = 0.02)	Reduced dyspnea post-exacerbation
Tan et al., 2019, RCT [34]	20-min mindful breathing session	Borg Dyspnea Scale	Dyspnea ↓ at 5 min (U = 233.5, *p* < 0.001); ↓ at 20 min (U = 232.0, *p* = 0.001)	Rapid and significant reduction in dyspnea
Tschenett et al., 2025, pilot RCT [35]	Digital MBI (daily audio via app)	CAT, CRQ-SAS	Momentary dyspnea ↓ (ηp^2^ = 0.70, *p* < 0.001); CRQ-SAS improved (ηp^2^ = 0.14, *p* = 0.004)	Reduced dyspnea and improved functioning

Abbreviations: ACQ-5: Asthma Control Questionnaire (5-item), ASI-3: Anxiety Sensitivity Index-3, CAT: COPD Assessment Test, CI: Confidence Interval, COPD SE Scale: COPD Self-Efficacy Scale, CRQ-SAS: Chronic Respiratory Questionnaire—Self-Administered Standardized Format, EQ-5D: EuroQol-5 Dimensions Questionnaire, FMI: Freiburg Mindfulness Inventory, FFMQ: Five Facet Mindfulness Questionnaire, HADS: Hospital Anxiety and Depression Scale, MBI: Mindfulness-Based Intervention, MBSR: Mindfulness-Based Stress Reduction, PHLMS: Philadelphia Mindfulness Scale, *p*: *p*-value, PSS-10: Perceived Stress Scale (10-item version), RCT: Randomized Controlled Trial, SCS: Self-Compassion Scale, SGRQ: St. George’s Respiratory Questionnaire, U: Mann–Whitney U test, ηp^2^: partial eta squared.

## Data Availability

All data are included in this study.

## Appendix A

PUBMED

“Respiration Disorders”[Mesh] OR “Pulmonary Disease, Chronic Obstructive”[Mesh] OR “Chronic Obstructive Pulmonary Diseases” OR “COPD” OR “Chronic Obstructive Lung Disease” OR “Chronic Airflow Obstruction” AND “Mindfulness”[Mesh] OR “Mindfulness Meditation” OR “Meditation, Mindfulness” OR “Mindfulness Meditations” OR meditation OR MBCT OR MBSR OR MBCR OR “Virtual Reality”[MeSH Terms] OR “Avatar”[Mesh] OR “virtual reality” OR “VR” OR “Humanoid Avatar” OR “Psychosocial Intervention”[Mesh] OR “Intervention, Psychosocial” OR “Psychological Intervention” OR “Psychological Interventions”

Scopus

“Respiration Disorders” OR “Pulmonary Disease, Chronic Obstructive” OR “Chronic Obstructive Pulmonary Diseases” OR “COPD” OR “Chronic Obstructive Lung Disease” OR “Chronic Airflow Obstruction” AND “Mindfulness” OR “Mindfulness Meditation” OR “Meditation, Mindfulness” OR “Mindfulness Meditations” OR meditation OR MBCT OR MBSR OR MBCR OR “Virtual Reality” OR “Avatar” OR “virtual reality” OR “VR” OR “Humanoid Avatar” OR “Psychosocial Intervention” OR “Intervention, Psychosocial” OR “Psychological Intervention” OR “Psychological Interventions”

WOS

“Respiration Disorders” OR “Pulmonary Disease, Chronic Obstructive” OR “Chronic Obstructive Pulmonary Diseases” OR “COPD” OR “Chronic Obstructive Lung Disease” OR “Chronic Airflow Obstruction” AND “Mindfulness” OR “Mindfulness Meditation” OR “Meditation, Mindfulness” OR “Mindfulness Meditations” OR meditation OR MBCT OR MBSR OR MBCR OR “Virtual Reality” OR “Avatar” OR “virtual reality” OR “VR” OR “Humanoid Avatar” OR “Psychosocial Intervention” OR “Intervention, Psychosocial” OR “Psychological Intervention” OR “Psychological Interventions”
